# Diagnostic accuracy of probe-based confocal laser endomicroscopy and tissue sampling by endoscopic retrograde cholangiopancreatography in indeterminate biliary strictures: a meta‑analysis

**DOI:** 10.1038/s41598-022-11385-4

**Published:** 2022-05-04

**Authors:** Junjie Mi, Xiaofang Han, Rong Wang, Ruijun Ma, Danyu Zhao

**Affiliations:** 1 Digestive Endoscopy Center, Shanxi Provincial People’s Hospital, No. 29, Shuang ta si Street, Taiyuan, China; 2 Reproductive Medicine, Shanxi Provincial People’s Hospital, Taiyuan, China

**Keywords:** Biliary tract disease, Biliary tract disease

## Abstract

Probe-based confocal laser endomicroscopy (pCLE), also known as optical biopsy, is a new endoscopic technique that provides real-time magnification of 1000 × microscopic tissue information to diagnose indeterminate biliary strictures. Tissue sampling by endoscopic retrograde cholangiopancreatography (ERCP) is routinely performed to evaluate indeterminate biliary strictures. To evaluate the accuracy of pCLE and tissue sampling by ERCP in the diagnosis of indeterminate biliary strictures, 18 articles were included from 2008 to 2021 through Embase, PubMed, Web of Science, and Cochrane library databases. The summary estimates for the pCLE diagnosis of indeterminate biliary strictures were: sensitivity 0.88 (95% confidence interval (CI), 0.84–0.91); specificity 0.79 (95% CI 0.74–0.83); and Diagnostic Odds Ratio (DOR) 24.63 (95% CI 15.76–38.48). The summary estimates for tissue sampling by ERCP diagnosis for indeterminate biliary strictures were: sensitivity 0.54 (95% CI 0.49–0.59); specificity 0.96 (95% CI 0.94–0.98); and DOR 11.31 (95% CI 3.90–32.82). The area under the sROC curve of pCLE diagnosis of indeterminate biliary strictures is 0.90 higher than 0.65 of tissue sampling by ERCP. The pCLE is a better approach than tissue sampling by ERCP for the diagnosis of indeterminate biliary strictures by providing real-time microscopic images of the bile ducts.

## Introduction

Biliary strictures are a common disease of the biliary system. The treatment and prognosis of benign and malignant biliary strictures vary greatly, so it is especially important to distinguish between benign and malignant biliary strictures. Although there are various advanced imaging techniques such as peroral choledochoscopy (POCS) and biopsy techniques such as endoscopic ultrasonography guided fine needle aspiration (EUS-FNA) and endoscopic retrograde cholangiopancreatography (ERCP) biopsy and cytology brush, accurate diagnosis of indeterminate biliary strictures still has challenge^1^. Cholangiocarcinoma is the most common malignant tumor causing malignant strictures of the bile duct, and most tumors grow along the bile duct wall instead of radially to form masses^2^. Therefore, cholangiocarcinoma is usually at an advanced stage when diagnosed, and the survival rate of patients is low. At the same time, as many as 15% of the suspected hilar cholangiocarcinoma were confirmed to be benign by surgical histopathology^2,3^. Thus, an urgent clinical need exists to improve the diagnostic accuracy of indeterminate biliary strictures so that malignant tumors can be detected as early as possible, improving the prognosis for patients and reducing unnecessary surgeries.

Probe-based confocal laser endomicroscopy (pCLE) is a new diagnostic endoscopy approach that provides real-time microscopic tissue information. The pCLE enables low-energy laser light to be focused and illuminated through its inner small hole to produce a tissue image, which improves the accuracy of targeted biopsy and realizes instant optical biopsy^4^. The pCLE has been used to assess the histopathology of the gastrointestinal tract in vivo^5,6^. The accuracy of pCLE in the diagnosis of gastrointestinal tumors has been demonstrated in previous studies^7,8^. To date, pCLE has been studied in a variety of digestive diseases, including ulcerative colitis, colorectal polyps, pancreatic disease, and Barrett's esophagus^9–12^. In the same situation, pCLE can also enter the intrahepatic and extrahepatic bile ducts to provide real-time enlarged tissue information for bile duct lesions. The pCLE thus improves the accuracy of diagnosing indeterminate biliary strictures. With the development of pCLE technology and the improvement of the diagnostic classification of indeterminate biliary strictures, a series of studies have been published on the diagnosis of indeterminate biliary strictures in pCLE^13–30^. The present study will perform a meta-analysis to assess the accuracy of pCLE in diagnosing indeterminate biliary strictures and to compare the accuracy of pCLE and ERCP with brush cytology and intraductal biopsy in diagnosing indeterminate biliary strictures.

## Results

### Search results and quality assessment

**Figure 1:**
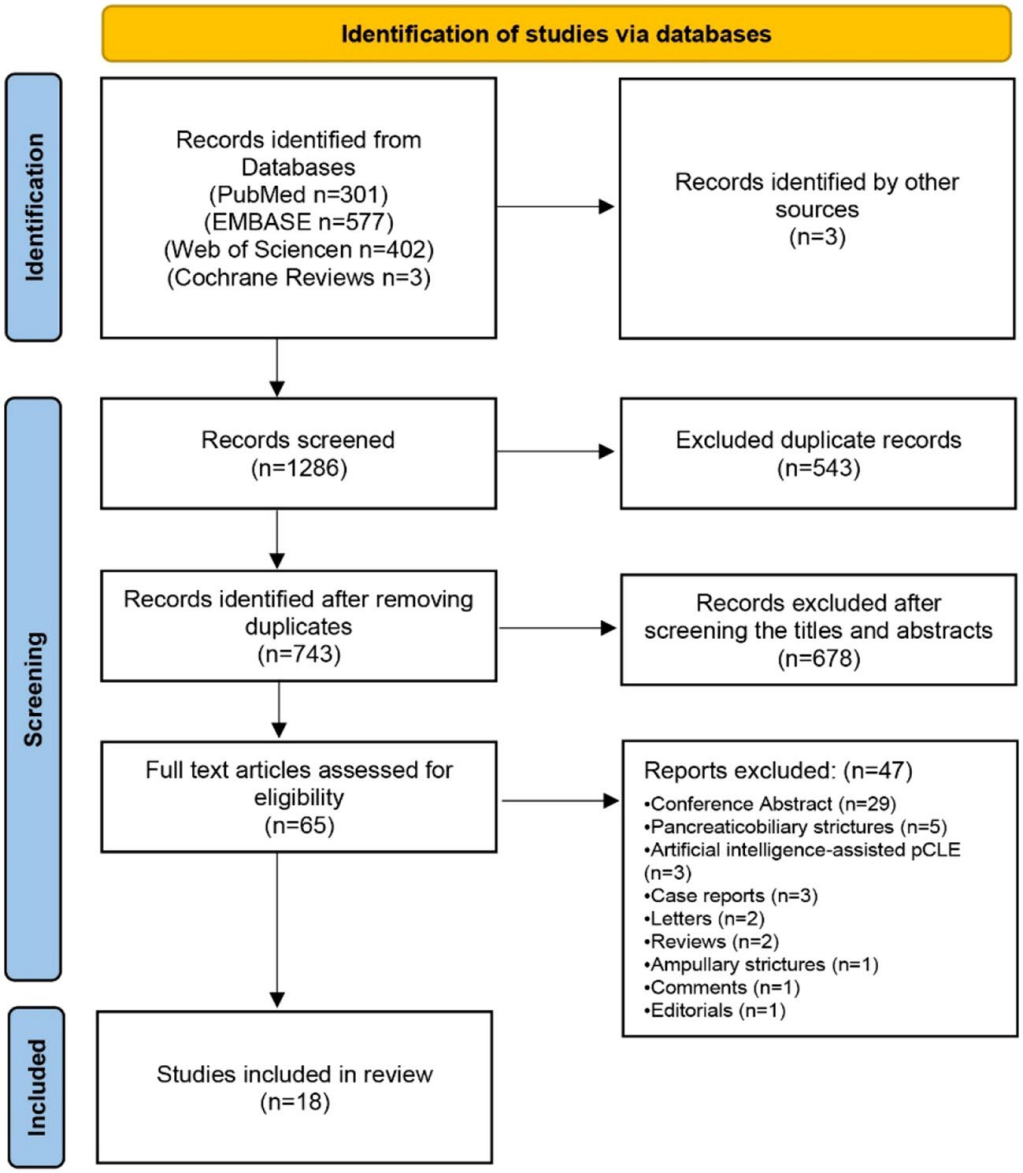
PRISMA flow chart.

**Figure 2:**
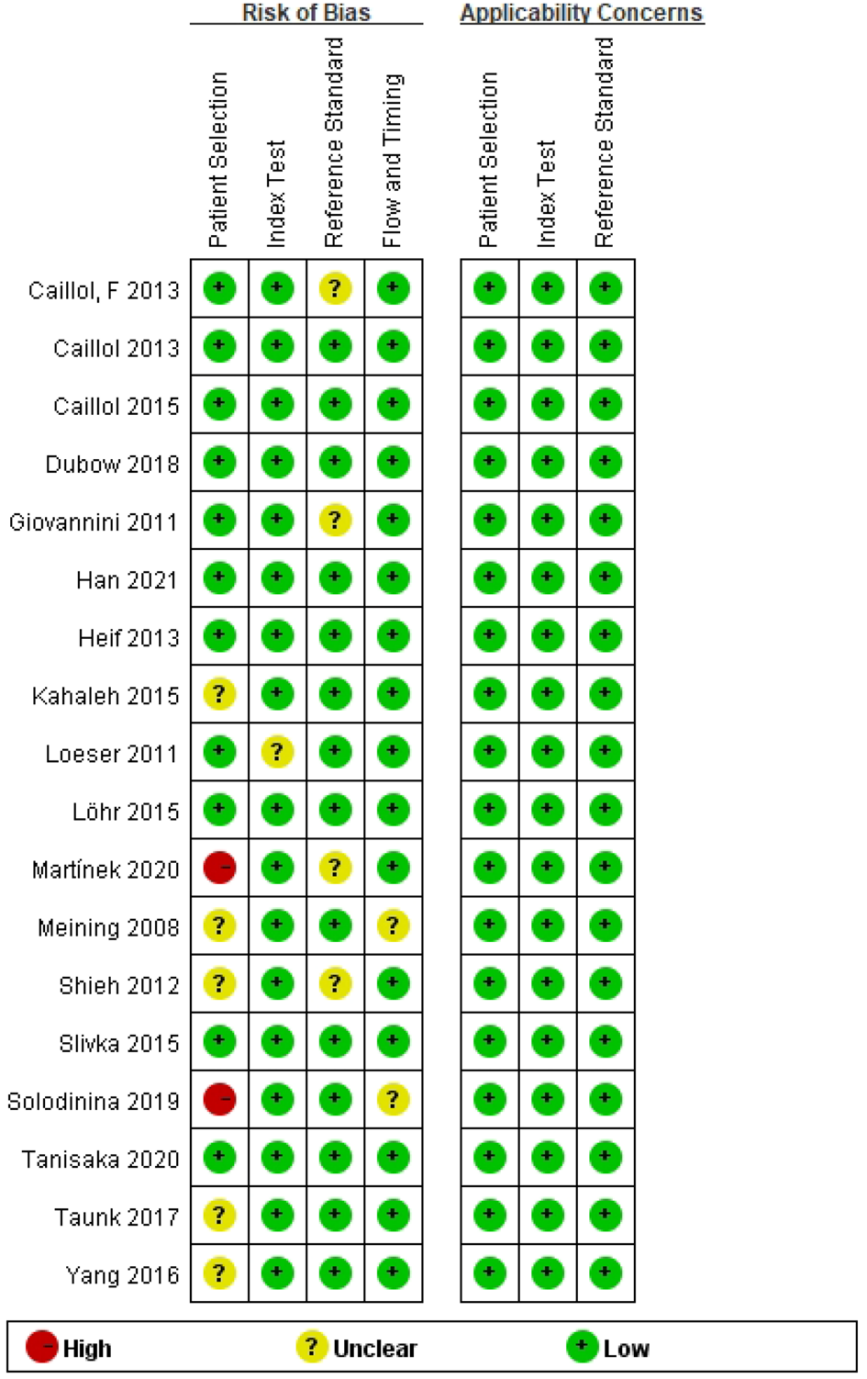
The quality of included studies and the risk of bias.

Searching using the preset search strategy resulted in 1286 records in Fig. 1. After eliminating duplicate records, there were 743 records left. 678 records were excluded immediately after a review of titles and abstracts. After reading the full text of the remaining records, 47 records were further excluded due to various reasons. Three records were added after a manual review of the references of the 15 remaining articles retrieved. Eighteen studies published between 2008 and 2021 were finally included in the meta-analysis^13–30^. In total, there were 750 lesions reported by 18 studies, 12 prospective and 6 retrospectives in Table 1. There were 1286 records for this study in Supplementary Information. The quality of the included studies was assessed using the quality assessment of diagnostic accuracy studies (QUADAS) assessment tool as detailed in Fig. 2.

**Table 1:** Characteristics of the selected studies.

Author	Year	Mean age (years)	Female (%)	Country	Method	Design	Diagnostic classification	Follow-up (month)	Center	Mini probe	Presence of primary sclerosing cholangitis
Meining	2008	61.3	42.9	Germany	Cholangioscopy	Retrospective	Not used	> 9	Single	CholangioFlex	NO
Loeser	2011	Unclear	Unclear	USA	Catheter or cholangioscopy	Retrospective	Not used	7–12	Single	Gastroflex	NO
Giovannini	2011	62.3	37.8	France	Catheter	Retrospective	Not used	Unclear	Single	CholangioFlex	NO
Shieh	2012	Unclear	Unclear	USA	Catheter	Retrospective	Not used	> 12	Single	Gastroflex	NO
Heif	2013	54.0	26.7	USA	Catheter or cholangioscopy	Retrospective	Miami	12–36	Single	CholangioFlex	YES
Caillol	2013	Unclear	Unclear	France	Catheter or cholangioscopy	Retrospective	Miami	12	Single	CholangioFlex	NO
Caillol	2013	66.0	42.6	France	Catheter	Prospective	Miami	12	Single	CholangioFlex	NO
Caillol	2015	67.0	45.9	France	Catheter	Retrospective	Miami	12	Single	CholangioFlex	NO
Kahaleh	2015	Unclear	Unclear	USA	Catheter	Retrospective	Paris	Unclear	Single	CholangioFlex	NO
Slivka	2015	64.5	46.0	USA	Catheter	Prospective	Miami and Paris	6–12	Multiple	CholangioFlex	NO
Löhr	2015	56.0	53.3	Sweden	Catheter or cholangioscopy	Prospective	Miami and Paris	> 12	Single	Gastroflex	NO
Yang	2016	66.0	45.1	USA	Catheter or cholangioscopy	Retrospective	Miami and Paris	> 12	Multiple	CholangioFlex	NO
Taunk	2017	64.5	19.0	USA	Catheter or cholangioscopy	Retrospective	Miami and Paris	12	Single	CholangioFlex	NO
Dubow	2018	56.0	36.0	USA	Unclear	Retrospective	Miami and Paris	> 6	Single	CholangioFlex	NO
Solodinina	2019	66.0	42.8	Russia	Cholangioscopy	Retrospective	Unclear	12–48	Single	CholangioFlex	NO
Tanisaka	2020	71.0	13.3	Japan	Cholangioscopy	Prospective	Miami	> 12	Single	CholangioFlex	NO
Martínek	2020	61.0	52.2	Czech	Cholangioscopy	Prospective	Miami and Paris	10	Multiple	CholangioFlex	NO
Han	2021	49.0	41.0	USA	Catheter or cholangioscopy	Prospective	Miami and Paris	> 12	Multiple	CholangioFlex	YES

### Diagnostic accuracy of pCLE in indeterminate biliary strictures

**Figure 3:**
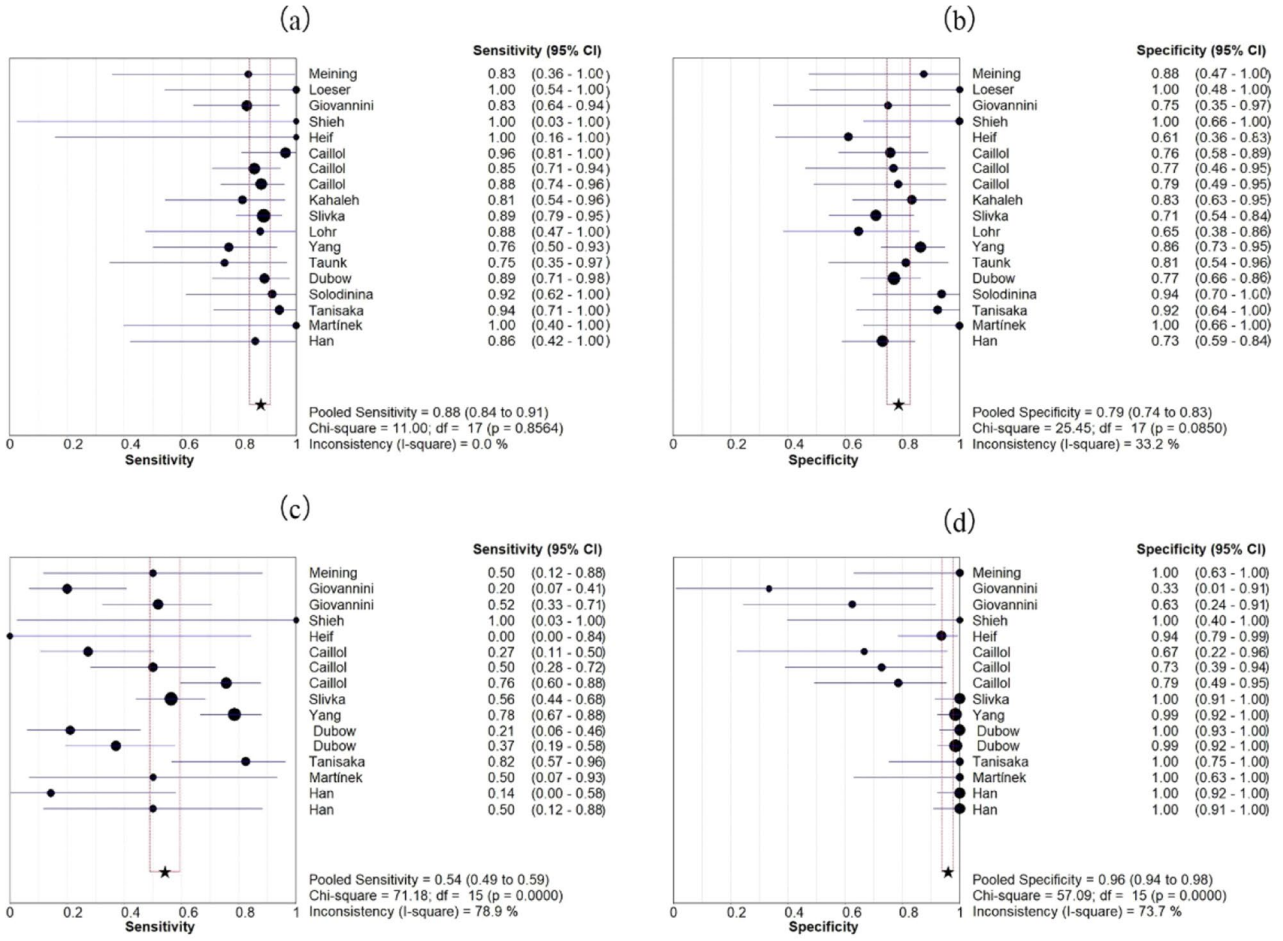
Forest plot of sensitivity and specificity of pCLE (**a**,**b**) and tissue sampling by ERCP (**c**,**d**) in diagnosing indeterminate biliary strictures.

**Figure 4:**
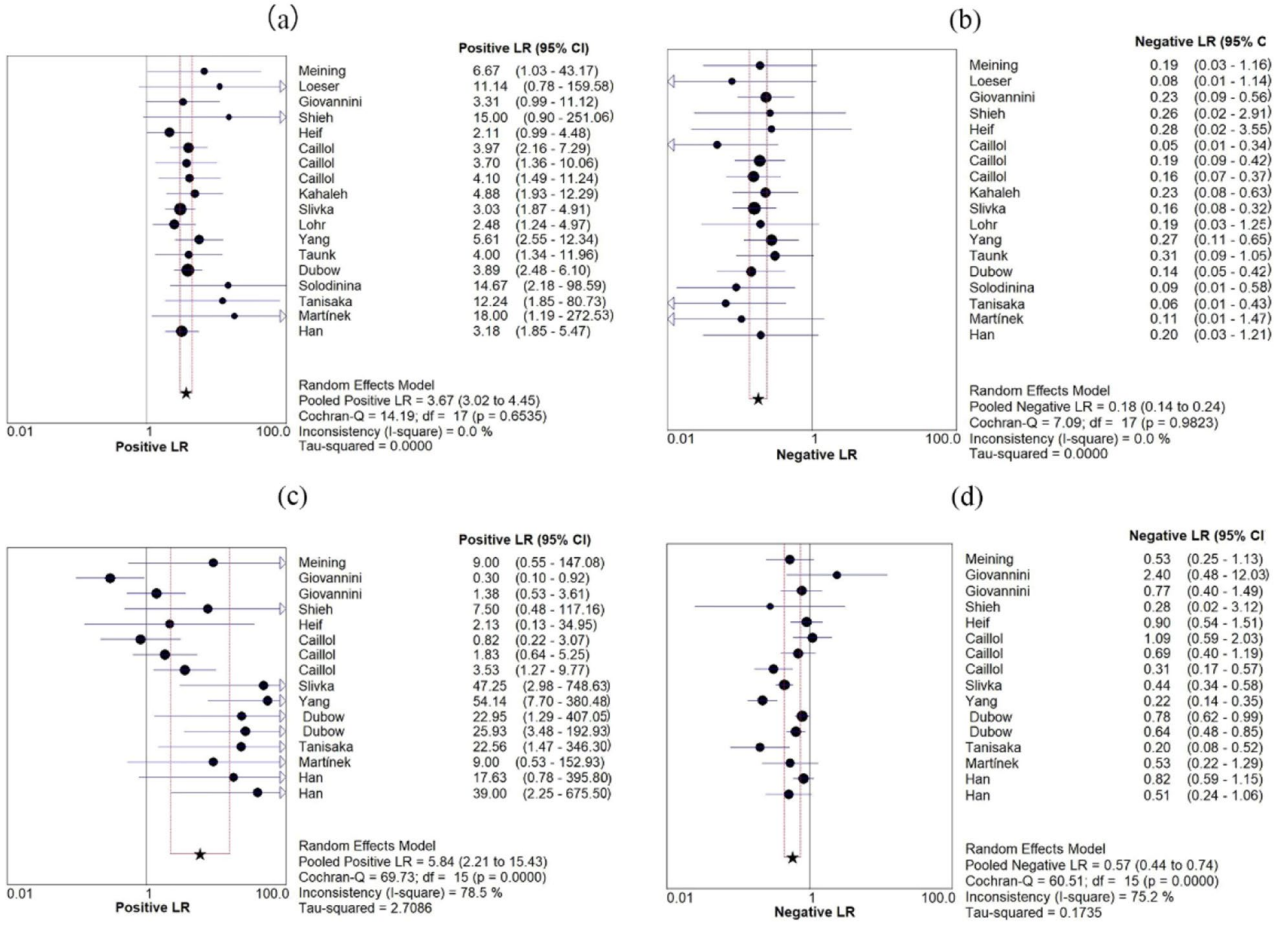
Forest plot of PLR and NLR of pCLE (**a**,**b**) and tissue sampling by ERCP (**c**,**d**) in diagnosing indeterminate biliary strictures.

**Figure 5:**
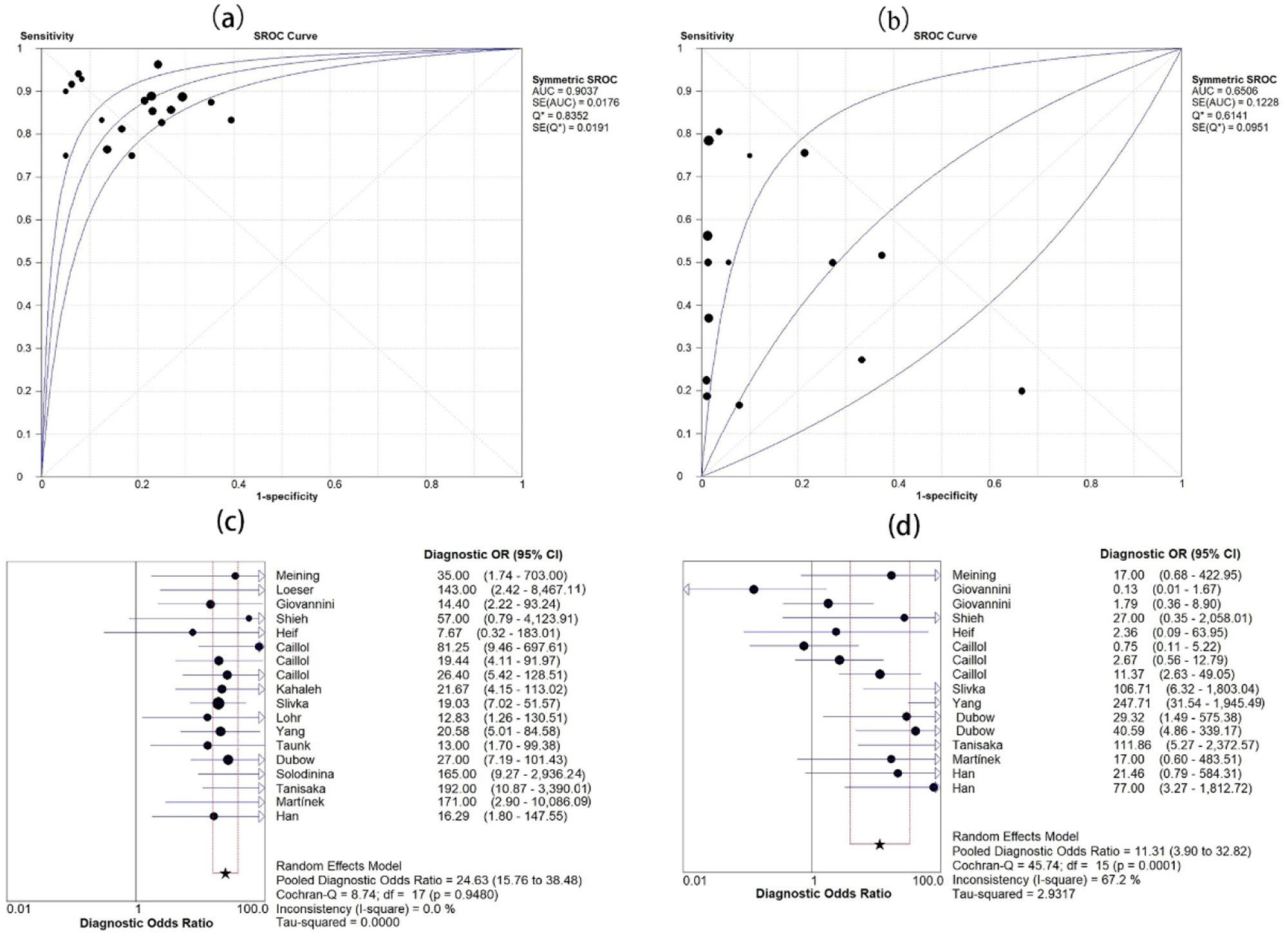
SROC plot and DOR of pCLE (**a**,**c**) and tissue sampling by ERCP (**b**,**d**) in diagnosing indeterminate biliary strictures.

Eighteen studies evaluated the accuracy of pCLE diagnosis of indeterminate biliary strictures comprising a total of 750 lesions. Figure 3 shows the sensitivity and specificity of pCLE in the diagnosis of indeterminate biliary strictures. The sensitivity range was 0.75–1.00, and the specificity range was 0.61–1.00. The pooled sensitivity and specificity were 0.88 (95% CI 0.84–0.91) and 0.79 (95% CI 0.74–0.83), respectively. No significant heterogeneity was found in terms of sensitivity and specificity (I^2^ = 0.0%, I^2^ = 33.2%). An indicator of test accuracy, the Diagnostic Odds Ratio (DOR), combines sensitivity and specificity data into one. DOR equals the value of positive likelihood ratio (PLR) divided by the value of negative likelihood ratio (NLR). A higher value indicates that the test has better diagnostic capabilities. The combined PLR, NLR, and DOR were 3.67 (95% CI 3.02–4.45), 0.18 (95% CI 0.14–0.24, Fig. 4) and 24.63 (95% CI 15.76–38.48, Fig. 5), respectively, and PLR, NLR, and DOR all had I^2^ values of 0.0%, which indicated that there is no significant heterogeneity. Figure 5 shows the sROC curve of pCLE in the diagnosis of indeterminate biliary strictures. The area under the sROC curve (AUC) is a method of assessing the diagnostic ability, and it represents the total diagnostic ability. The higher the AUC value, the better the diagnostic ability, and a value of 1.0 indicates that the diagnosis has a near-perfect diagnostic ability. The pCLE diagnosis of indeterminate biliary strictures had a fairly high accuracy rate with an AUC value of 0.90 (SE = 0.02). Another way to evaluate the diagnostic performance of the sROC curve is the index Q. The current index Q value was 0.84, which also shows that the diagnostic accuracy is high.

### Diagnostic accuracy of ERCP with brush cytology and intraductal biopsy in indeterminate biliary strictures

A total of 16 studies including 784 lesions evaluated the accuracy of tissue sampling by ERCP in the diagnosis of indeterminate biliary strictures, among which intraductal biopsy was 9 articles, brush cytology was 4 articles, brush cytology and intraductal biopsy were 3 articles. Figure 3 shows the sensitivity and specificity of ERCP with brush cytology and intraductal biopsy in the diagnosis of indeterminate biliary strictures. The pooled sensitivity and specificity were 0.54 (95% CI 0.49–0.59) and 0.96 (95% CI 0.94–0.98), respectively. We found significant heterogeneity in terms of sensitivity and specificity (I^2^ = 78.9%, I^2^ = 73.7%). The combined PLR, NLR, and DOR were 5.84 (95% CI 2.21–15.43), 0.57 (95% CI 0.44–0.74, Fig. 4), and 11.31 (95% CI 3.90–32.82, Fig. 5), respectively, and PLR, NLR, and DOR had I^2^ values of 78.5%, 75.2%, and 67.2%, respectively, which indicated that there is significant heterogeneity. The ERCP with brush cytology and intraductal biopsy diagnosis of indeterminate biliary strictures had a poor accuracy with an AUC value of 0.65 (SE = 0.09) in Fig. 5. The accuracy of pCLE in diagnosing indeterminate biliary strictures was better than ERCP with brush cytology and intraductal biopsy, with a significant difference with 95% confidence regions overlapping.

### Subgroup analyses

We conducted subgroups based on the characteristics of pCLE to diagnose indeterminate biliary strictures. To understand the pCLE diagnosis of indeterminate biliary strictures between different subgroups, we conducted a subgroup analysis and compared whether there are significant statistical differences. The subgroup analysis is shown in Table 2. The subgroups are as follows: diagnostic classification, method, mini probe, country, study design, primary sclerosing cholangitis, follow-up, study center, no. of the lesion, stricture locations. There are two diagnostic classifications for pCLE diagnostic indeterminate biliary strictures, the Paris classification and the Miami classification. Although the sensitivity of the Paris classification in diagnosing indeterminate biliary strictures was lower than that of the Miami classification in the subgroup analysis (0.75 versus 0.88), the specificity of the Paris classification was higher than that of the Miami classification (0.85 versus 0.77). Depending on the mode of entry of pCLE into the bile ducts, there are two subgroups: the cholangioscope subgroup and the catheter subgroup. The cholangioscope subgroup has better sensitivity and specificity for diagnosing indeterminate biliary strictures than the catheter subgroup (0.92 versus 0.86, 0.93 versus 0.78). Two common Mini probes for diagnosing indeterminate biliary strictures in pCLE are the Gastroflex and CholangioFlex. The sensitivity and specificity of the diagnosis of indeterminate biliary strictures in the Gastroflex subgroup are better than those in the CholangioFlex subgroup (0.93 versus 0.87, 0.80 versus 0.78). Primary sclerosing cholangitis is a common cause of indeterminate biliary strictures. The sensitivity of the primary sclerosing cholangitis subgroup to diagnose indeterminate biliary strictures is higher than that of the subgroup without primary sclerosing cholangitis (0.88 versus 0.87), but the specificity is lower than that of the subgroup without primary sclerosing cholangitis (0.70 versus 0.80). The primary sclerosing cholangitis subgroup has a low specificity in the diagnosis of indeterminate biliary strictures, which may be due to severe inflammation of the bile ducts leading to high false positives. 95% confidence regions have overlap in all different subgroup comparisons, indicating that the differences between subgroups are not statistically significant.

**Table 2:** Subgroup analysis of diagnostic indices (with 95% confidence interval).

Subgroup	No. of studies	Sensitivity pooled (%)	Specificity pooled (%)	PLR pooled	NLR pooled	DOR pooled	AUC	Index Q
**Diagnostic classification**								
Miami classification	6	88.2 (81.6–93.1)	77.6 (68.5–85.1)	4.27 (2.83–6.46)	0.15 (0.09–0.25)	29.45 (13.56–63.95)	0.89	0.82
Paris classification	2	75.0 (53.3–90.2)	85.0 (70.2–94.3)	4.91 (2.27–10.63)	0.30 (0.15–0.60)	17.12 (4.93–59.48)	Unavailable	Unavailable
**Method**								
Cholangioscope	4	92.3 (79.1–98.4)	93.5 (82.1–98.6)	11.99 (4.31–33.35)	0.10 (0.04–0.27)	112.27 (24.41–516.43)	0.97	0.92
Catheter	6	86.4 (80.9–90.9)	78.0 (69.0–85.4)	3.57 (2.51–5.08)	0.19 (0.13–0.27)	20.24 (10.99–37.27)	0.90	0.83
**Mini probe**								
Gastroflex	3	93.3 (68.1–99.8)	80.6 (62.5–92.5)	3.97 (1.91–8.24)	0.16 (0.05–0.58)	25.06 (4.11–152.74)	0.92	0.86
CholangioFlex	15	87.4 (83.3–90.8)	78.6 (74.2–82.6)	4.13 (3.31–5.16)	0.17 (0.12–0.23)	25.52 (16.49–39.48)	0.90	0.83
**Country**								
Europe	8	88.1 (82.2–92.6)	79.7 (71.3–86.5)	4.28 (2.95–6.19)	0.14 (0.08–0.22)	31.11 (15.37–62.99)	0.92	0.85
USA	9	86.5 (80.0–91.4)	77.8 (72.4–82.5)	7.78 (2.93–4.88)	0.20 (0.13–0.29)	20.64 (11.96–35.63)	0.89	0.82
**Study design**								
Prospective	6	88.5 (82.2–93.2)	75.2 (67.3–82.0)	3.63 (2.58–5.10)	0.15 (0.09–0.25)	22.70 (11.59–44.47)	0.95	0.89
Retrospective	12	87.0 (81.4–91.4)	80.8 (75.5–85.3)	4.50 (3.42–5.93)	0.17 (0.12–0.25)	27.45 (15.89–47.44)	0.90	0.83
**Presence of primary sclerosing cholangitis**								
YES	2	88.9 (51.8–99.7)	70.0 (57.9–80.4)	2.78 (1.79–4.30)	0.22 (0.05–0.96)	12.68 (2.14–75.22)	Unavailable	Unavailable
NO	16	87.6 (83.6–91.0)	80.6 (76.0–84.7)	4,24 (3.38–5.32)	0.16 (0.12–0.22)	26.88 (17.41–41.50)	0.90	0.83
**Follow-up (month)**								
> 12 Months	11	87.8 (82.2–92.2)	78.8 (73.1–83.7)	4.37 (3.24–5.90)	0.16 (0.11–0.25)	27.16 (14.96–49.31)	0.90	0.83
< 12 Months	7	87.4 (81.2–92.1)	78.8 (71.8–84.8)	3.87 (2.85–5.24)	0.17 (0.11–0.26)	23.73 (12.97–43.41)	0.91	0.85
**Study center**								
Single	14	88.0 (83.2–91.8)	79.2 (73.8–83.9)	4.36 (3.34–5.69)	0.15 (0.11–0.22)	28.21 (16.65–47.80)	0.90	0.84
Multiple	4	86.9 (78.6–92.8)	78.1 (70.5–84.5)	3.67 (2.57–5.23)	0.19 (0.11–0.32)	20.69 (10.08–42.46)	0.90	0.83
**No. of lesion**								
> 50	7	87.9 (83.0–91.8)	76.8 (71.2–81.7)	3.70 (2.88–4.75)	0.16 (0.11–0.24)	24.30 (14.36–41.12)	0.88	0.82
< 50	11	87.2 (79.4–92.8)	82.5 (75.3–88.4)	5.22 (3.47–7.85)	0.17 (0.11–0.28)	27.74 (13.50–57.00)	0.91	0.84
**Stricture locations**								
Common bile duct	4	80.0 (64.4–90.9)	84.6 (69.5–94.1)	3.51 (1.31–9.42)	0.34 (0.09–1.25)	12.12 (1.68–87.35)	0.88	0.81
Extrahepatic bile ducts	2	92.3 (64.0–99.8)	87.1 (70.2–96.4)	6.36 (0.98–41.18)	0.14 (0.03–0.64)	49.34 (3.45–706.35)	Unavailable	Unavailable

### Publication bias and sensitivity analysis

**Figure 6:**
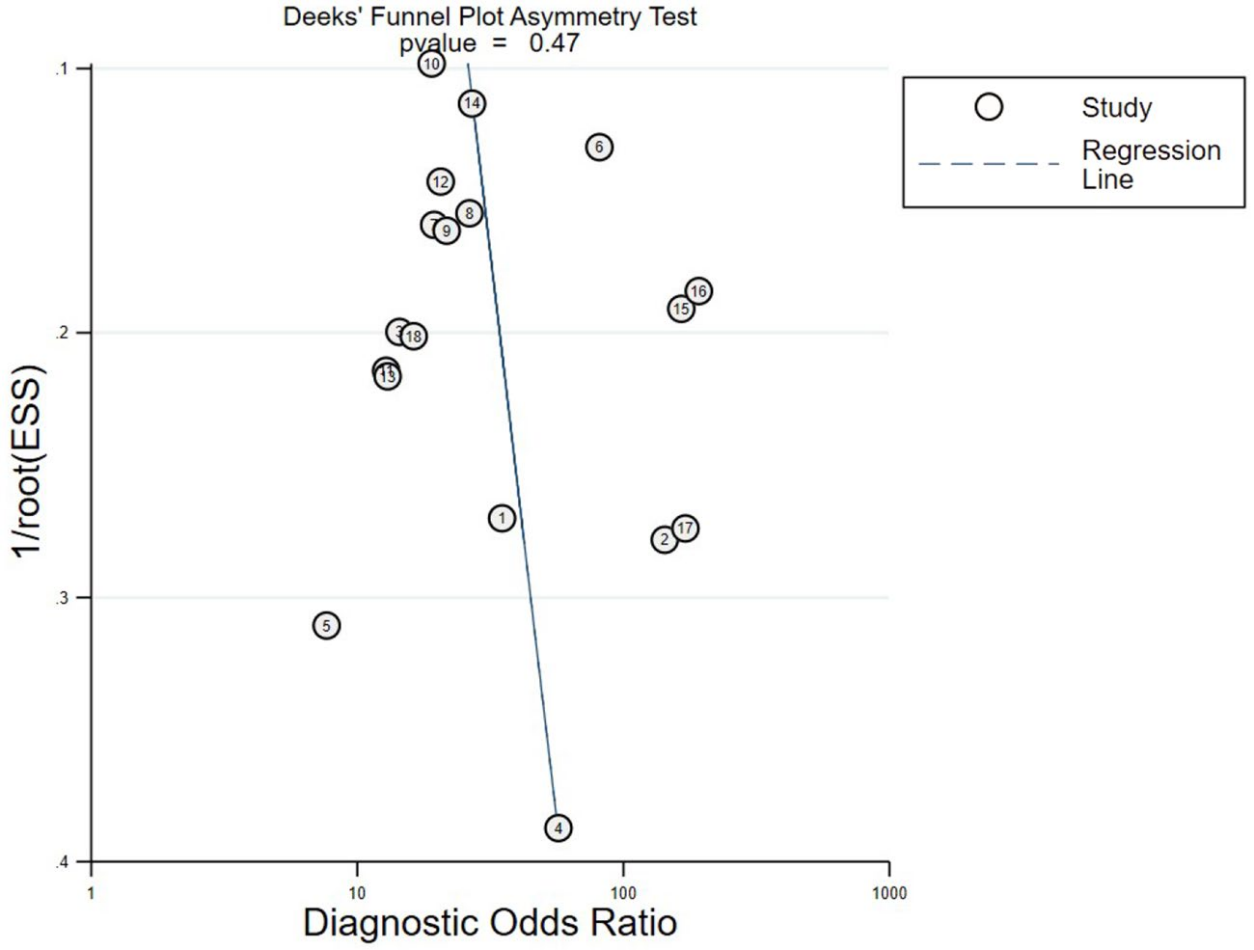
Funnel plot for the evaluation of potential publication bias of selected studies.

Deek’s funnel plot was used to analyze the potential publication bias of the meta-analysis. Deek’s test showed a value of 0.47 (95% CI − 4.95 to 10.32), and these suggested no possibility of publication bias in Fig. 6. There was also no significant threshold effect by Spearman correlation coefficient on pCLE diagnosis of indeterminate biliary strictures (Spearman correlation coefficient: − 0.07; P = 0.77). For sensitivity analysis, each study was excluded in turn, and the AUC was recalculated for the remaining articles. Yang's study had the greatest impact on the results of the meta-analysis, taking the AUC from 0.9037 to 0.9092 (+ 0.6%)^15^. The results of the sensitivity analysis showed that the diagnostic efficacy of pCLE for indeterminate biliary strictures remained stable at high levels.

## Discussion

The identification of benign and malignant biliary strictures is critical to patient treatment and prognosis. Early diagnosis of benign biliary strictures can reduce unnecessary surgery and costs, and early detection of bile duct malignancies allows for early surgical intervention to improve surgical cure rates and improve patient prognosis. However, due to the particularity of the anatomical structure of the bile duct and the way of tumor growth, the current diagnostic methods still have insufficient sensitivity and accuracy. Therefore, accurate diagnosis of indeterminate biliary strictures is still a major clinical problem.

The diagnosis of biliary strictures is a complex and multiple approach. EUS-FNA is a method for obtaining biopsy tissue from biliary lesions and has 87% sensitivity and 87% accuracy for the diagnosis of indeterminate biliary strictures in a prospective single-center trial^31^. However, the shortcoming of this method is that it is limited to intrahepatic biliary strictures or proximal extrahepatic biliary strictures. Single-person operation of POCS allows insertion of the extrahepatic bile duct for direct visualization of the lesion and obtaining a targeted biopsy. In a meta-analysis of single-operator POCS including 335 patients, the sensitivity and specificity of visual representation for diagnosing indeterminate biliary strictures were 90% and 87%, respectively, whereas the sensitivity and specificity of single-operator POCS biopsy for diagnosing indeterminate biliary strictures were 69% and 98%, respectively^32^. However, differences in the interobserver agreement were found in various studies, which may be due to the lack of a standard consensus for the diagnosis of indeterminate biliary strictures in POCS^33,34^. In addition, POCS may have difficulty accessing the intrahepatic bile duct and obtaining sufficient tissue samples to confirm a malignancy, but pCLE allows adequate visualization of intrahepatic bile duct strictures because of its small diameter^14^. pCLE has a high accuracy rate in both intrahepatic and extrahepatic biliary strictures due to its fine outer diameter and access to the intrahepatic bile ducts, and there is no statistical difference between intrahepatic (100%) and extrahepatic biliary strictures (86%)^17^. In addition, for indeterminate biliary strictures where the mass is not visible on the image, it is difficult to obtain the specimen by biopsy, but pCLE can obtain a high diagnostic accuracy of 79% and an negative predictive value (NPV) of 100%^30^. pCLE is a new imaging technique capable of providing real-time microscopic tissue information in vivo to obtain images of the GI epithelium and sub epithelium at 1000 magnification during endoscopy. Real-time high-resolution histological diagnosis of mucosa and submucosa tissue structure can achieve the purpose of real-time optical biopsy^35^. As an emerging endoscopic technique, pCLE is gradually spreading worldwide, and the recent American Society for Gastrointestinal Endoscopy guidelines on the management of biliary tract tumors mention pCLE as a useful alternative to existing diagnostic workup^36^. In many studies, pCLE has high sensitivity and accuracy for the diagnosis of indeterminate biliary strictures.

The meta-analysis showed that pCLE as a diagnostic tool for indeterminate biliary strictures had a pooled sensitivity of 0.88 and a pooled specificity of 0.79. We reported that the index Q value of sROC was 0.84 and AUC was 0.90, indicating that the overall diagnostic accuracy is very high. The range of DOR is from 0 to infinity, which can be used as an overall assessment of the accuracy of diagnosis. When the DOR value is 1.0, the test is an invalid diagnostic method^37^. Our study reported a pooled DOR of 24.63, which also illustrates the high overall diagnostic accuracy. Because PLR and NLR are more easily applied in the clinical setting, they were used as an indicator to assess diagnostic accuracy in the current meta-analysis. A PLR value of 3.67 indicated that patients with malignant biliary strictures were approximately three times more likely to be positive compared to patients without malignant biliary strictures. In contrast, the NLR is found to be 0.18, which means that if the pCLE test is negative, the patient has about a 18% chance of having malignant biliary strictures. Therefore, based on current data, PLR and NLR may be used as valuable tools for diagnosing uncertain biliary strictures in pCLE in the future.

We noted differences in diagnostic accuracy between subgroups. The diagnosis of pCLE for indeterminate biliary strictures has a Miami classification and a Paris classification, and we compared the diagnostic accuracy of the Miami and Paris classifications in a subgroup analysis. The Miami classification of pCLE diagnostic indeterminate biliary strictures was developed in 2012 and tested by a multicenter study^38^. The image features of Miami classification are shown in Table 3. In order to improve the specificity of pCLE diagnosis of indeterminate biliary strictures and reduce the false positive rate related to chronic inflammation, Caillol proposed the Paris classification related to benign inflammation changes^28^. The Paris classification is based on the Miami classification with the addition of 4 image features of benign inflammatory stenosis as shown in Table 3. The use of the Paris classification to evaluate the diagnostic accuracy of pCLE for indeterminate biliary strictures showed that the Paris classification can improve the specificity of the diagnosis of indeterminate biliary strictures^24,29^. Similar results were obtained by using the Paris classification in the subgroup of the present study, improving the specificity of the diagnosis from 77.6 to 85.0%. In future studies, each criterion of the Paris classification scheme for diagnosis of indeterminate biliary strictures needs to be evaluated and appropriate thresholds need to be set. In addition, the thickness of the reticular structure in the Paris classification also needs to be quantified.

**Table 3:** Both the Miami-criteria and Paris-criteria for the diagnosis of indeterminate biliary strictures in pCLE.

Tumorous bile duct	Normal bile duct	Inflammatory bile duct
Thick white bands (> 20 μm)	Reticular network of thin dark branching bands (< 20 μm)	Vascular congestion
Thick dark bands (> 40 μm)	Vessels (< 20 μm)	Dark granular patterns with scales
Dark clumps	Light gray background	Increased inter-glandular space
Epithelium		Thickened reticular structure

Because pCLE can reach the biliary strictures by a catheter or a cholangioscopy, we compared the diagnostic accuracy of the two approaches in a subgroup analysis. The pCLE may reach the biliary strictures more accurately by direct visualization with a cholangioscopy, while the pCLE can only reach the biliary strictures by catheter under fluoroscopic guidance. The cholangioscopy can adjust the angle in the bile duct to make the mini probe to the site of interest diagnose indeterminate biliary strictures in pCLE and obtain a high accuracy of 93.3%^17^. There are similar results in the present study, where the sensitivity and specificity of pCLE for the diagnosis of indeterminate biliary strictures in the cholangioscopy subgroup were higher at 92.3% and 93.5%, respectively. Additionally, pCLE operators' skills should be improved through training, as highly skilled operators are able to achieve high accuracy^16,18,39^.

In most pCLE diagnostic studies on indeterminate biliary strictures, the CholangioFlex mini probe was used, which has an outer diameter of less than 1 mm and a resolution of 3.5 mm. However, some studies have used the GastroFlex mini probe, which increases the number of optical fibers and has better resolution and image quality^19,22,23^. Good image quality achieved 100% accuracy while poor image accuracy was only 79%, indicating that good image quality can significantly improve accuracy^28^. In addition, the Gastroflex probe had a larger outer diameter but no reduction in cannulation rate^19^. Usually, the larger the outer diameter of the probe, the lower the rate of cannulation. In Shieh's study, the high rate of cannulation with the Gastroflex probe may be due to the operator's being a skilled endoscopist^19^. More accurate diagnoses were obtained in some studies using the Gastroflex probe, but the small number of Gastroflex probes used in these studies will require further confirmation in studies with large sample sizes. In the subgroup analysis of this study, the sensitivity and specificity of pCLE in the subgroup of using the GastroFlex mini probe for the diagnosis of indeterminate biliary strictures reached 93.3% and 80.6%. However, the diagnostic performance of the GastroFlex mini-probe will need to be further clarified by multi-center prospective studies in the future.

A subgroup analysis was performed to determine the diagnostic ability of pCLE for different stricture locations. The sensitivity and specificity of pCLE in diagnosing indeterminate biliary strictures were high for both extrahepatic and common bile duct strictures. The diagnosis of indeterminate biliary strictures in the intrahepatic bile ducts was of concern. However, due to the lack of data on intrahepatic biliary strictures, meta-analysis was not performed, and more studies are needed in the future to determine the accuracy of pCLE in the diagnosis of intrahepatic biliary strictures. In addition, if some objective diagnostic methods such as artificial intelligence and signal-to-noise ratio are used, it may improve the sensitivity and specificity of pCLE to diagnose indeterminate biliary strictures^20^. Based on the pattern of tumor growth, cholangiocarcinoma is classified into three subtypes: mass-forming, periductal infiltration, and intraductal growth, with the mass-forming type being the most frequent and the intraductal growth type being the least common. There is a lack of study of pCLE diagnosing indeterminate biliary strictures based on tumour growth patterns, and further study is needed to determine if pCLE is accurate in diagnosing indeterminate biliary strictures based on different tumour growth patterns. In sum, although the data on the accuracy of CLE was promising, a high level of operator expertise and standardized training is required. There was no data on operator learning curves, although one study showed that inexperienced operators achieved 83% accuracy after three weeks of professional training^36^. Therefore, the training of endoscopists in pCLE has been strengthened to improve the reliability of pCLE imaging and the consistency of image interpretation in order to better apply this new technology.

Brush cytology and intraductal biopsy by ERCP are routinely performed to evaluate indeterminate biliary strictures^40^. We performed a meta-analysis of brush cytology and intraductal biopsies by ERCP. The meta-analysis showed that ERCP with brush cytology and intraductal biopsy as a diagnostic tool for indeterminate biliary strictures has a pooled sensitivity of 0.54 and a good specificity of 0.96. We reported that the index Q value of sROC is 0.61 and AUC is 0.65, indicating that the overall diagnostic accuracy is poor. There is no overlap between the 95% AUC confidence intervals of pCLE and brush cytology and intraductal biopsy in the diagnosis of indeterminate biliary strictures, indicating that there are significant differences between the two groups. The sensitivity of 0.54 for ERCP with brush cytology and intraductal biopsy is indeed disappointing. The reasons for the low sensitivity could be tumor-associated fibrosis, submucosal spread, or bile duct compression by external lesions. Desmoplastic tumors have a relatively small number of cells and are very firm, making sampling very difficult. Another reason for the poor diagnosis of ERCP with brush cytology and intraductal biopsy is the random sampling of tissues. The pCLE also allows for a more accurate diagnosis by locating different biliary strictures in real time. ERCP with brush cytology and intraductal biopsy combined with pCLE can obtain a more accurate diagnosis^18,30^.

This study has several limitations. First, as most of the included studies were retrospective studies, and there was potential selective bias needing further prospective studies to confirm. Secondly, the poor quality of some of the included studies may affect the results of the meta-analysis. Third, some patients in some of the included studies were followed up rather than pathology as the gold standard.

In conclusion, pCLE is a reliable and accurate method for diagnosing indeterminate biliary strictures, especially when reaching the biliary strictures by cholangioscopy. However, pCLE is expensive compared to common ERCP, and future studies are needed to confirm the cost-effectiveness. With the development of diagnostic classifications and advances in technology, pCLE will improve the accuracy of diagnosing indeterminate biliary strictures. The pCLE has the potential to overcome the limitations inherent in tissue sampling by ERCP by providing real-time microscopic images of the bile ducts to make an accurate diagnosis of indeterminate biliary strictures.

## Materials and methods

### Search strategy and study selection

The study was conducted in accordance with the PRISMA (Preferred Reporting Project for Systematic Reviews and Meta-Analysis) guidelines. The PRISMA checklist for this study was in the Supplementary Information. The deadline for searching PubMed, Web of Science, Cochrane Library, Embase is October 2021. Search method: (“probe-based confocal laser endomicroscopy” or “confocal laser scanning microscopy” or “confocal microscopy” or “confocal endomicroscopy” or “endomicroscopy” or “pCLE” or “CLE”)AND(“bile duct carcinoma” or “bile duct” or “bile duct neoplasms” or “biliary strictures” or “indeterminate biliary strictures”). The bibliographies of the retrieved articles were carefully screened for potentially relevant study and the search was not limited to any specific language. Data searching was performed independently by two reviewers (Mi and Han), and disagreements were resolved by consensus.

The studies included in the meta-analysis met the following criteria: (1) The included studies were all published proposing the diagnosis of pCLE on indeterminate biliary strictures; (2) All studies included for the diagnosis of indeterminate biliary strictures had pathology and follow-up as the gold standard for final diagnosis; (3) The number of true positives (TP), false positives (FP), true negatives (TN) and false negatives (FN) can be obtained directly or indirectly in the study. Exclusion criteria: (1) Conference abstracts, letters to editors, reviews, case reports, comments, editorials; (2) pCLE diagnosis of pancreaticobiliary strictures; (3) animal or pediatric studies; (4) pCLE diagnosis of ampullary strictures; (5) artificial intelligence-assisted pCLE (Supplementary Information 1).

### Data extraction and quality assessment

Data extraction for each selected study was performed independently by two reviewers (Mi and Wang), and disagreements were resolved by consensus. The extracted content included the general information of the study, such as year of publication, first author, age, sex, country, diagnostic classification, mode of entry into the bile duct, follow-up time, study design, number of research centers, type of microprobe, and presence of primary sclerosing cholangitis. To assess the quality and potential bias of the studies, QUADAS was applied to evaluate the quality of the included studies. Each eligible study was independently evaluated by two authors (Ma and Zhao), and differences were resolved through discussions with the third author (Mi).

### Statistical analyses

Estimates of diagnostic accuracy for pooled studies, including sensitivity, specificity, PLR, NLR, and DOR were calculated. The primary outcome of the present meta-analysis was to assess the pooled results of the accuracy of the pCLE diagnostic indeterminate biliary strictures, with the secondary aim of assessing the pooled results of the accuracy of the tissue sampling by ERCP diagnostic indeterminate biliary strictures. The pooled results used a random effects model, as the random effects model (DerSimonian–Laird method) provides more conservative results. In the present meta-analysis, the Cochrane *Q* test was used to assess heterogeneity between studies, with heterogeneity between studies rather than chance expressed as a percentage (I^2^). More than 50% of the I^2^ were considered to be significantly heterogeneous. The sROC using the method of Moses and colleagues is a comprehensive indicator to evaluate the accuracy of diagnosis. sROC is a graph of the functional relationship between the true positive rate and the false positive rate based on a linear regression model. Overlapping 95% CI for the given values were determined as qualitative methods indicating no significant differences. To explore the accuracy of pCLE diagnostic indeterminate biliary strictures in different subgroups, a subgroup analysis was performed according to the following: year of publication, first author, country, diagnostic classification, mode of entry into the bile duct, follow-up time, study design, number of research centers, type of microprobe, stricture locations and presence of primary sclerosing cholangitis. The present meta-analysis used Deek’s test and funnel plot analysis for publication bias.

Meta-DiSc software version 1.4 was used to test the diagnostic accuracy, including sensitivity, specificity, PLR, NLR, DOR, sROC, and subgroups. The Spearman’s correlation coefficient was used to assess the threshold effect using Meta-DiSc software version 1.4. STATA software version 16.0 was used to analyze publication bias. The quality of the included studies was assessed using Review Manager5.3 software. To assess the robustness of the synthesized results, sensitivity analyses will be conducted. The significance level was measured at P < 0.05.

## Supplementary Information

Supplementary Information 1.

Supplementary Information 2.

Supplementary Information 3.

Supplementary Information 4.
